# Assessing the stability of azopolymer nanotopography during live-cell fluorescence imaging

**DOI:** 10.3389/fbioe.2024.1409735

**Published:** 2024-08-13

**Authors:** Mona H. Abdelrahman, Jerry Shen, Nicholas C. Fisher, Wolfgang Losert, John T. Fourkas

**Affiliations:** ^1^ Department of Chemistry and Biochemistry, University of Maryland, College Park, MD, United States; ^2^ Pharmaceutical Analytical Chemistry Department, Faculty of Pharmacy, Ain Shams University, Cairo, Egypt; ^3^ Department of Physics, University of Maryland, College Park, MD, United States; ^4^ Institute for Physical Science and Technology, University of Maryland, College Park, MD, United States; ^5^ Maryland Quantum Materials Center, University of Maryland, College Park, MD, United States

**Keywords:** live-cell imaging, fluorescence microscopy, reprogrammable nanotopography, azopolymers, esotaxis, cytoskeletal dynamics, 2-photon microscopy

## Abstract

**Introduction:**

Photomodifiable azopolymer nanotopographies represent a powerful means of assessing how cells respond to rapid changes in the local microenvironment. However, previous studies have suggested that azopolymers are readily photomodified under typical fluorescence imaging conditions over much of the visible spectrum. Here we assess the stability of azopolymer nanoridges under 1-photon and 2-photon imaging over a broad range of wavelengths.

**Methods:**

Azopolymer nanoridges were created via microtransfer molding of master structures that were created using interference lithography. The effects of exposure to a broad range of wavelengths of light polarized parallel to the ridges were assessed on both a spinning-disk confocal microscope and a 2-photon fluorescence microscope. Experiments with live *Dictyostelium discoideum* cells were also performed using alternating cycles of 514-nm light for photomodification and 561-nm light for fluorescence imaging.

**Results and Discussion:**

We find that for both 1-photon and 2-photon imaging, only a limited range of wavelengths of light leads to photomodification of the azopolymer nanotopography. These results indicate that nondestructive 1-photon and 2-photon fluorescence imaging can be performed over a considerably broader range of wavelengths than would be suggested by previous research.

## 1 Introduction

Cells live in dynamic microenvironments, and have the capability to respond and adjust their functions accordingly (Gattazzo et al., 2014). *In vivo*, the extracellular matrix plays a pivotal role in governing cellular behaviors by providing mechanical support and mechanical cues to cells, significantly influencing functions such as metabolism and transport processes. A comprehensive understanding of the effective regulation of cell behaviors, encompassing aspects such as adhesion, orientation, migration, and differentiation on artificial surfaces, is vital in biomedical engineering, and in the life sciences in general (Chen et al., 2017).

Photomodifiable nanotopographies provide a unique means of assessing how cells respond to rapid changes in their physical microenvironment (Chen et al., 2017). Studying the responses of cells to photoinduced changes in such nanotopographies can provide insights into how to design surfaces that can elicit specific cellular behaviors. Such an understanding can contribute to the development of materials and substrates that can be used in cellular biophysics to gain insights into cell-signaling pathways and to recapitulate the dynamic changes of the natural microenvironment (Bettinger et al., 2009). This information is also valuable in applications including medical device development and tissue engineering, as well as in a wide range of therapeutic interventions (McNamara et al., 2010). Surface engineering can be exploited further to enhance or control drug release based on cellular responses, which has implications for the development of more effective and better targeted drug-delivery systems (Finbloom et al., 2023).

Azobenzene serves as the parent molecule for a group of compounds with the distinctive ability to isomerize reversibly between a stable *trans* form and a metastable *cis* form upon absorption of light within a particular wavelength range (Yager and Barrett, 2006). Polymers incorporating azobenzene moieties are referred to as azopolymers. Azopolymers have been used in a wide range of applications, including the creation of photomodifiable nanotopographies. These materials can be used to create a diverse range of surfaces via inscription of nanotopography through methods such as embossing complex laser patterns onto thin films via a conventional confocal microscope (Viswanathan et al., 1999; Priimagi and Shevchenko, 2014; Sun et al., 2018; Audia et al., 2020). Additionally, nanotopographies previously fabricated through various lithographic techniques can be replicated in azopolymers via stamping (Rianna et al., 2016). The resulting azopolymer surfaces, e.g., nanopillars (Jo et al., 2020) and micropillars (Oscurato et al., 2017; De Martino et al., 2020), can then be photomodified, including in the presence of cells.

The photomodification of azopolymers stems from the geometric rearrangement of azobenzene molecules upon exposure to light. Linearly-polarized light induces preferential alignment of the azobenzene chromophores that arises from fluidization that occurs along the direction of the light polarization (Jo et al., 2020). If nanoridges are aligned parallel to the direction of the light polarization, the polymer will expand in that direction, leading to buckling. Conversely, circularly-polarized light induces broadening of nanoridges, because the transition dipoles of the azopolymer molecules tend to point along the beam axis, and hence the polymer backbone tends to point perpendicular to the beam axis.

A number of studies have been conducted to investigate the behavior of cells on photomodifiable nanotopographies composed of poly (Disperse Red 1 methacrylate), pDR1m. The majority of this research has focused on fluorescence imaging of fixed, stained cells for which the nanotopography was photomodified while the cells were alive. For instance, fixed breast-cancer epithelial cells and kidney epithelial cells were investigated on pDR1m pillars that had been photomodified (Puliafito et al., 2019). The response of stem cells to photomodification of a flat pDR1m surface was investigated using fixing and staining, and revealed changes in alignment, cell shape, and mechanical properties within 1–4 h after pattern inscription, with alterations arising from focal adhesions and extending into chromatin (Cimmino et al., 2022). The mechanical properties of fibroblasts on azopolymer substrates with various nanotopographic patterns have also been studied using fixing and staining. That work revealed that cells orient along linear patterns, impacting cytoskeletal structures such as actin stress fibers, and also demonstrated that cells recognize nanotopographic patterns and transfer mechanical information via the cytoskeleton, leading to nuclear deformation that is linked to differing mechanical properties in the nuclear region (Rianna et al., 2015). Photopatterned azopolymer films have also been demonstrated to influence the *in vitro* behavior of stem cells, such as aggregation and preferential elongation (Salvatore et al., 2020).

Viability tests have demonstrated the biocompatibility of pDR1m surfaces, and qualitative and quantitative analyses indicated the significant influence of grating-like nanopatterns on cell nuclei and cytoskeletal actin filaments (Salvatore et al., 2020). In these studies, live-cell imaging was conducted using CellTracker Deep Red dye to stain living cells. Confocal microscopy was employed to capture images and videos, with the dye excited at 633 nm and emission collected in the wavelength range of 650–745 nm. According to the authors, the choice of dye was crucial to avoid photomodification of the surface during imaging by ensuring that the absorption wavelengths of the azopolymer and the fluorescent dye did not overlap (Rianna et al., 2016). Additionally, the dynamic modulation of membrane curvature has been explored in live cells through the engineering of light-responsive, azobenzene-based polymer structures, demonstrating the ability to induce well-defined curvatures in adherent cells using green-light illumination while observing the resulting effects on actin dynamics. For fluorescence imaging, the authors employed a 0.1 mW of blue light, at an unspecified wavelength, without causing modification of the azopolymer (De Martino et al., 2020).

Here we explore the conditions under which live-cell fluorescence imaging can be performed without disrupting azopolymer nanotopographies. We find that such imaging is feasible on pDR1m nanotopographies across a broad range of wavelengths without inducing any alteration to the nanotopography itself. The most effective wavelength for inducing photoswitching between the *cis* and *trans* forms using linear absorption is approximately 530 nm, wherein both isomers exhibit absorption. We find that by controlling the frame rate and the laser power, it is possible to use even a laser at 561 nm for fluorescence imaging without inducing photomodification of pDR1m nanotopography. In the case of 2-photon fluorescence excitation, we find that wavelengths from 750 nm to 900 nm do not induce photomodification of the nanotopography. Our results demonstrate the potential for using pDR1m nanotopographies for fluorescence imaging of live cells with multiple fluorescence labels without inducing modification of the nanotopography.

## 2 Materials and methods

### 2.1 Molding nanoridges from the master pattern and preparing pDR1m replicas

A 35-mm-diameter silicon master with distinct regions of nanoridge patterns with different spacings was fabricated using interference lithography. The average width of the nanoridges in all of the patterns was 400 nm. Prior to molding, the master was subjected to oxygen plasma for 30 s at 200 mTorr using a plasma cleaner (PDC-32G, Harrick). The master was then placed in a desiccator and exposed to (tridecafluoro-1, 1, 2, 2-tetrahydrooctyl)methyldichlorosilane (SIT8172.0, Gelest) vapor for 1 h. The objective of the silanization process was to reduce the surface energy of the master, ensuring a smooth release of cured polydimethylsiloxane (PDMS) molds. The molding procedure followed a previously established method (Sun et al., 2018).

Hard PDMS (h-PDMS) was prepared by mixing 1.7 g of 7.0%–8.0% trimethyloxy-terminated vinylmethylsiloxane-dimethylsiloxane copolymer (VDT-731, Gelest), 9 μL of platinum catalyst (SIP6831.2, Gelest), 0.05 g of 2,4,6,8-tetramethyl-2,4,6,8-tetravinylcyclotetrasiloxane as a modulator (87,927, Sigma-Aldrich), 0.5 g of (25%–30% methylhydrosiloxane)-(dimethylsiloxane) copolymer (HMS-301, Gelest), and 1 g of hexane as a solvent. This mixture was spin-coated at 1,000 rpm for 40 s on the master surface, followed by room-temperature curing for 2 h and additional curing in an oven at 60°C for 1 h. Sylgard 184 PDMS (Dow Corning) was prepared in a 10:1 mixture of the base monomer to the curing agent. Following three cycles of degassing and mixing, the Sylgard PDMS was poured onto the precured h-PDMS, creating a layer with an approximate thickness of 5 mm. The sample was kept in an oven at 60°C for 1 h, after which the PDMS mold was released from the master surface.

For preparing replicas, 1 in × 1 in pieces of glass slides and 22-mm-diameter coverslips with a thickness of 0.13–0.17 mm were sonicated in acetone for 10 min. This process was repeated in isopropyl alcohol and then in double-distilled, deionized water. The substrates were dried subsequently in an oven at 110°C for 1 h, and then were allowed to cool to room temperature before use. A cleaned substrate was treated with one drop of 2 wt% pDR1m (Sigma-Aldrich) in N,N-dimethyl formamide. The mold was placed gently over the substrate, and the combined assembly was kept in an oven at 55°C for 2 h to facilitate solvent evaporation. The mold was then released gently along the direction of the nanoridges, sprayed with N,N-dimethyl formamide, soaked in ethanol for 10 min in an ultrasound bath to remove any residual pDR1M, and allowed to dry at room temperature.

### 2.2 Photomodification of the nanoridges for reciprocity studies

The reciprocity of the photomodification process of the azopolymer nanoridges was investigated using a 532-nm laser for exposure. The beam passed through a half-wave plate, a polarizer, a quarter-wave plate, and a beam expander before being projected on a substrate containing pDR1m nanoridges that was mounted vertically. The fast axis of the quarter-wave plate was aligned at a 45° angle relative to the polarization of the laser beam, generating circularly polarized light.

The 1/*e* (electric field) beam diameter was measured by monitoring the beam intensity with different amounts of the beam blocked. A chopper was used to modulate the laser beam, which was focused onto the active area of a photodiode. Lock-in detection was employed at the chopping frequency. The data points produced a sigmoidal curve that was differentiated to obtain a Gaussian profile that was used to determine the 1/*e* ^2^ intensity diameter, which corresponds to the 1/*e* electric-field diameter.

To study reciprocity, different combinations of power values and exposure times were employed while maintaining a constant power–exposure-time product of 45 J. The highest power used was 500 mW, with an exposure time of 1.5 min, and the lowest power used was 7.8 mW, with an exposure time of 96 min. The corresponding irradiance values ranged from 376.8 mW/cm^2^ to 5.9 mW/cm^2^.

### 2.3 Photomodification of the azopolymer nanoridges with a spinning-disk microscope

Photomodification of the azopolymer nanoridges patterned on cleaned coverslips was conducted using a spinning-disk confocal fluorescence microscope (Perkin Elmer UltraView VoX) with a ×40 objective. The coverslip orientation was adjusted on the stage such that the direction of light polarization was parallel to the nanoridges. As expected in this configuration, the ridges underwent buckling. This behavior is attributed to the tendency of the azopolymers to fluidize and extend along the direction of light polarization (Jo et al., 2020).

Trials were conducted using the full range of lasers available on the microscope (Table 1) to investigate how each wavelength affected the features of the nanoridges. This investigation involved tailoring the power used, adjusting the exposure time and the scan rate, and modifying the time over which the camera acquired an image.

**TABLE 1:** Characteristics of the lasers used with the spinning-disk microscope in the studies reported here.

Wavelength (nm)	Maximum power (mW)
405	50
445	40
488	50
514	25
561	50
640	40

For each wavelength, we selected consistent imaging parameters that resemble the settings typically employed for imaging MCF10A epithelial cells, a model cell line that moves slowly and is suitable for imaging at a low frame rate. The constant conditions under investigation included scans performed at a rate of 0.1 frame/s, laser power adjusted to 4.5 mW for each wavelength, a 300-ms duration for the camera to acquire an image, and total irradiation time of 10 min. We conducted another round of imaging under conditions that are typical imaging parameters for faster moving cells, such as *Dictyostelium discoideum*, with an increased scan rate of 0.2 frame/s; in this case, the acquisition duration was 100 ms and the power was set to 2.5 mW. For each wavelength, the orientation of the ridges was adjusted to be parallel to the laser polarization, so that any photomodification would be manifested in the form of buckling, which can be discerned readily. Although the form of the photomodification depends on the laser polarization, the efficiency is expected to be independent of the polarization.

### 2.4 2-Photon imaging

The 2-photon excitation experiment was conducted using a multiphoton fluorescence microscope that has been described in detail previously (Farrer et al., 1999). The excitation source was a tunable Ti:sapphire oscillator (Chameleon Ultra II) with a repetition rate of 80 MHz and a pulse length of ∼150 fs. The laser beam is directed through the reflected-light illumination port of an inverted microscope to the back aperture of an oil-immersion, 1.45 NA, ×100, infinity-corrected microscope objective (Zeiss, α Plan-FLUAR), which the beam overfills.

The average laser power at the sample was set to 2.7 mW for each excitation wavelength tested, which is in the range typical powers used for fluorescence imaging. Each designated area was irradiated with the specified wavelength for 10 min using continuous raster-scans driven by galvanometric mirrors, at a rate of 0.5 frames/s and a frame size of approximately 53 μm × 51 μm. The light was polarized parallel to the ridges in all cases. After the exposure experiments, the coverslip was removed from the stage holder and rinsed with ethanol to remove any residual immersion oil. The areas irradiated at each wavelength were identified using an optical microscope prior to scanning-electron microscopy (SEM) imaging.

### 2.5 Scanning-electron microscopy

SEM imaging was performed using a fully computer-controlled microscope with a Schottky field-emission cathode in combination with a Xe plasma-focused ion-beam column (XEIA FEG SEM, TESCAN, Ltd.). The acceleration voltage was set to 10 kV, and the beam intensity was set to 10. Before imaging, the coverslips and/or glass slides featuring azopolymer nanoridge patterns were affixed on a 50 mm × 6 mm M4 specimen mount. The substrates were subsequently sputter-coated with Pt/Pd or Au at 20 mA for 20 s within an argon-plasma environment (Cressington sputter coater 108).

### 2.6 Image analysis

The average width of the ridges in each SEM image in the reciprocity studies was measured after exposure to a fixed dose of 532-nm light. To measure the ridge width accurately, each image was converted into black areas (representing the ridges) and white areas (representing the grooves) using Photoshop (Adobe). Width measurements were performed using ImageJ (http://imagej.nih.gov). The scale of each image was converted from pixels to micrometers based on the scale bar. Subsequently, each image was binarized by thresholding, followed by rotation if necessary to ensure that the ridges were perfectly horizontal, as determined by checking the coefficient of determination for each corresponding fitting line. Next, the upper and lower edges of each ridge were identified, and the corresponding line graphs were plotted. The data points were exported to Excel, where the width of each ridge was calculated by subtracting the *y* values for both edges at the same value of *x*, followed by computing the mean value per ridge. This procedure was repeated for five ridges per image. The average width and standard deviation value per image were determined from the data for these five ridges.

Root-mean-square (RMS) analyses were conducted on SEM images of the azopolymer nanoridges that had been exposed at the various wavelengths examined here under four distinct imaging conditions. The first two light-exposure experiments adhered to the standard imaging protocols for MCF10A cells, with durations of 10 and 30 min respectively. The third light-exposure experiment adhered to the typical imaging conditions for *D. discoideum* cells, and the fourth light-exposure experiment used high-scan-rate imaging parameters. The analysis involved processing each SEM image individually. Each image underwent binarization using ImageJ. Ridges within each image were eroded, skeletonized, and aligned horizontally by rotating the image based on the coefficient of determination derived from the corresponding line graph. Data from each line graph were exported to Excel. The mean position value of each ridge was determined and subtracted from the data. The RMS deviation from the mean value was then calculated. RMS deviation values were computed for each of the four nanoridges within each image. The mean RMS deviation value and standard deviation were calculated from the RMS deviations of the four nanoridges. The workflow for this procedure is shown in Supplementary Figure S1.

### 2.7 The response of *D. discoideum* cells to *in situ* photomodification of pDR1m nanoridges

Coverslips with azopolymer nanoridges were soaked in ethanol for at least 12 h, and were subsequently allowed to dry on the benchtop for 1 h. Next, the azopolymer substrates were glued to the bottom of 35-mm-diameter dishes containing boreholes (MatTek) using an elastomeric encapsulant (Dow Sylgard 164), which was allowed to solidify for at least 1 h.

All *D*. *discoideum*, pH-GFP, limE-RFP cells of AX2 background used in the experiments were cultured in HL5 medium and grown in suspension to reach a concentration of approximately 2.5 × 10^6^ cells/mL. The cells were tagged fluorescently with pH-limE for visualization. Mutant phenotypes were selected using 0.1% Hygromycin B. The fluorescently-labeled cells grown in suspension were subjected to electrofusion to form giant cells (Gerisch et al., 2013). The giant cells and any remaining unfused cells were subsequently plated onto textured surfaces at a density of 1.0 × 10^5^ cells/mL. After plating, the cells were incubated for 1 h to allow for the development of surface adhesion, and thereby actin waves, before imaging.

Imaging of the cells on azopolymer nanoridges was conducted using a spinning-disk confocal microscope (Perkin Elmer UltraView VoX) with a ×40 objective. Cells were imaged in both bright-field and fluorescence channels. The fluorescence channels alternated between a 561-nm laser and a 514-nm laser for multiple cycles, starting with the 561-nm laser. The 561-nm channel was employed for imaging, and the 514-nm channel was used for modifying the azopolymer nanoridges. The ridges were aligned to be parallel to the polarization of the 514-nm laser.

We started with the 561-nm laser set to a power of 3.5 mW, a frame rate of 0.2 frames/s, an exposure time of 50 msec, and an exposure duration of 10 min. Subsequently, the 514-nm laser was set to 2.5 mW, with a rate of 1 frame/s, an exposure time of 1 s, and an exposure duration of 5 min. In both cases, the time lapse was adjusted to 5 s/timepoint.

## 3 Results

### 3.1 Reciprocity in pDR1m photomodification with 532-nm, circularly-polarized light

The principle of reciprocity states that a light source, regardless of its irradiance (*I*), will produce equivalent levels of darkening in photographic images when exposed over a duration (*t*) such that the product *It* remains constant (Schwarzschild, 1900). In photographic films, it is well known that reciprocity breaks down when the irradiance is small enough, in a phenomenon known as low-intensity reciprocity failure (LIRF) (Katz, 1949). LIRF arises in a regime in which the dose–response curve for the film becomes sublinear; hence, longer exposures than those predicted from *It* are required when the irradiance is sufficiently small.

It is important to assess whether or not pDR1m exhibits LIRF under conditions typically used for the fluorescence imaging of live cells, and, if so, at what wavelengths. LIRF would be advantageous in this context, enabling the imaging of cells over longer durations without impacting the pDR1m nanotopography unintentionally. We therefore sought to examine how the irradiance and exposure duration affect pDR1m nanoridges. Azopolymer nanoridges on glass substrates were irradiated with circularly-polarized, 532-nm light at different power–exposure-time combinations to determine the conditions under which reciprocity holds. The highest power used was 500 mW, with an exposure time of 1.5 min. The lowest power used was 7.8 mW, with an exposure time of 96 min. The product of the power and the exposure time was kept constant at 45 J in all cases. The longest exposure time was chosen to exceed the duration of the irradiation in a typical live-cell fluorescence imaging experiment. This time scale is generally limited by photobleaching of the fluorophores and/or phototoxicity.

**FIGURE 1:**
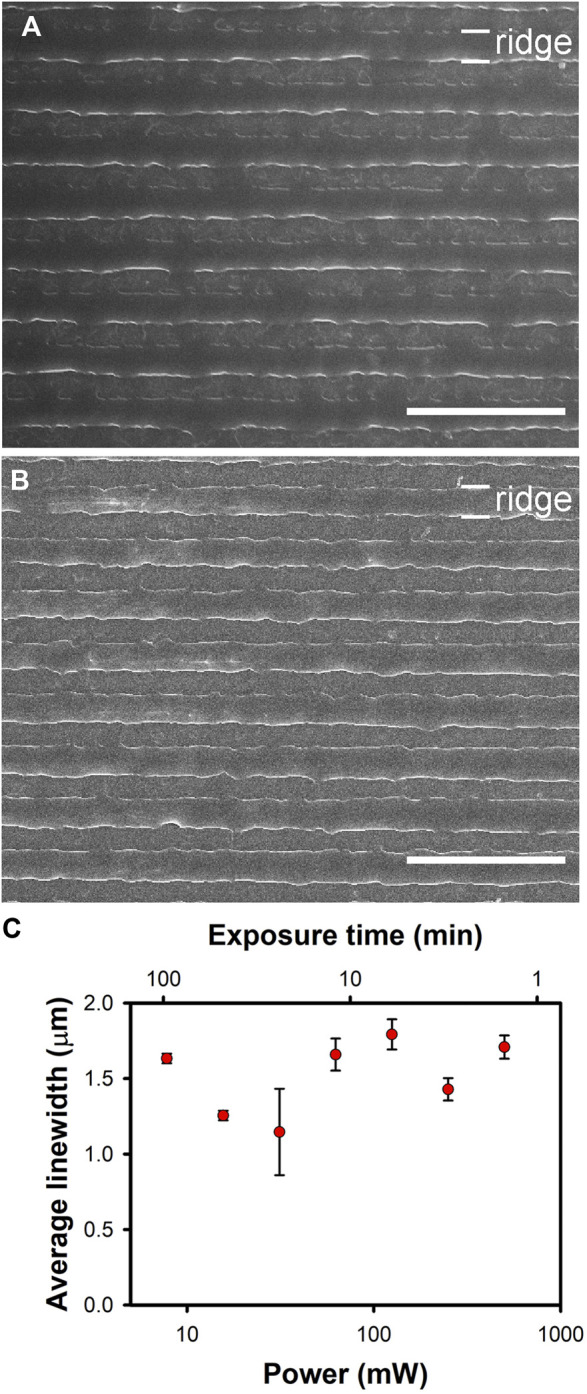
Broadening of azoploymer nanoridges upon exposure to fixed doses of 532-nm, circularly-polarized light with different powers and exposure times. The total dose in each case was 45 J. **(A)** Results for the highest laser power, 500 mW for 1.5 min. **(B)** Results for the lowest laser power, 7.8 mW for 96 min. The scale bars in **(A)** and **(B)** are 10 µm. **(C)** A plot depicting the relationship between the average ridge width in µm and the laser power. The average width values were determined as described in Section 2.6. Within experimental uncertainty, reciprocity is maintained over the entire range of powers explored.

SEM images of the azopolymer ridges following exposure to the highest and the lowest powers of the circularly-polarized light at the specified exposure times are shown in Figures 1A, B. The average width of the ridges for different exposure powers is depicted in Figure 1C. The pristine nanoridges have an average width of 400 nm. Upon exposure to circularly-polarized, 532-nm light at the fixed dose chosen, the average width of the ridges increases to roughly 1.5 μm. There is a spread of roughly ±20% in the average widths under the different exposure conditions, but there is no consistent trend in these deviations. Thus, we believe that deviations represent sample-to-sample variations, and that reciprocity remains in effect across the entire range of powers and exposure times examined. Although this outcome is not ideal from a live-cell imaging standpoint, it is encouraging that no unexpected effects are observed at high laser powers.

### 3.2 1-Photon irradiation studies over a broad range of wavelengths

**FIGURE 2:**
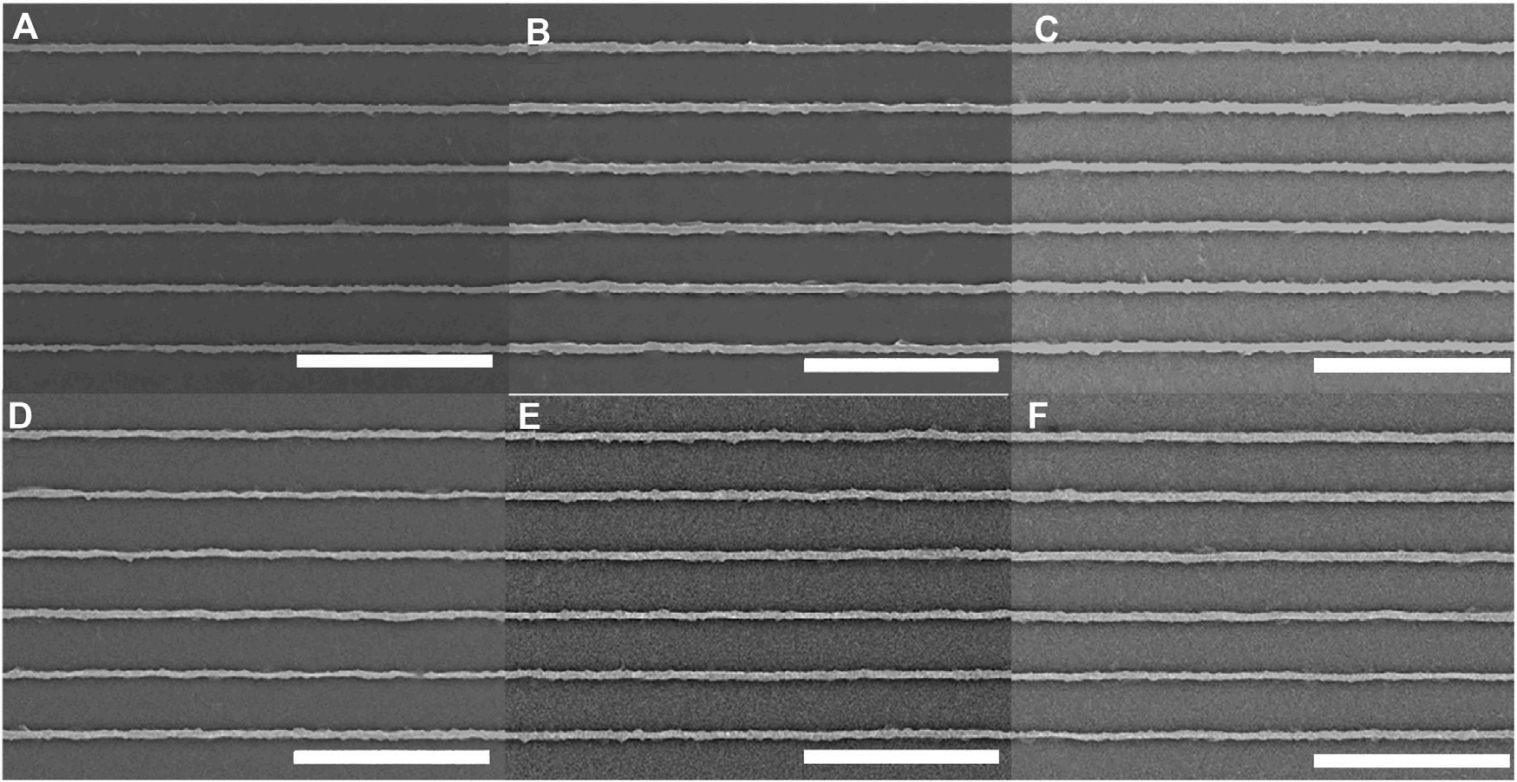
Azopolymer ridges exposed for 10 min to light at wavelengths of **(A)** 405 nm, **(B)** 440 nm, **(C)** 488 nm, **(D)** 514 nm, **(E)** 561 nm and **(F)** 640 nm, on a spinning-disk confocal microscope under constant exposure conditions that are typical for live-cell imaging. All scans were performed at a rate of 1 frame/10 s, the laser power at each wavelength was 4.5 mW, and the image-acquisition time was 300 ms. The ridges are the lighter regions. All scale bars are 10 µm.

We used a spinning-disk confocal microscope to study the effect of each available laser wavelength on the pDR1m nanoridges. For each wavelength, we employed consistent imaging parameters. First, we tested the typical parameters for imaging slow-moving, MCF10A epithelial cells. Scans were performed at a rate of 0.1 frame/s, the laser power was adjusted to 4.5 mW at each wavelength, the acquisition time was 300 ms, and the total exposure time was 10 min. SEMs of the nanoridges were acquired after exposure to 405-nm (Figure 2A), 440-nm (Figure 2B), 488-nm (Figure 2C), 514-nm (Figure 2D), 561-nm (Figure 2E), and 640-nm (Figure 2F) lasers polarized parallel to the nanoridges. In all cases the nanoridges did not exhibit any substantial waviness following exposure. These results were quantified by calculating the RMS deviation of individual nanoridges from SEM images. All RMS values were found to be in the range of 0.040 ± 0.006 μm (Supplementary Figure S2). The samples that underwent 405-nm and 640-nm exposure had the smallest RMS deviations, which were approximately 0.035 μm, with all of the other exposure wavelengths leading to a somewhat larger RMS deviation of approximately 0.045 μm as well as a greater standard deviation, based on measurements for four different ridges. For comparison, the RMS deviation for unexposed ridges was 0.041 ± 0.003 μm. This deviation is a consequence of the second-generation mold used to produce the azopolymer nanoridges, rather than of the azopolymer itself.

Further investigations were conducted using identical imaging parameters, but with an extended exposure time of 30 min (Supplementary Figure S3). SEM analysis revealed no discernible impact on the ridges following exposure at any wavelength, except for a slight waviness observed after exposure to 514-nm light. The RMS deviation values remained consistent across all wavelengths at 0.042 ± 0.003 μm, except for the 514-nm exposure, which exhibited a deviation of 0.052 ± 0.001 μm (Supplementary Figure S4). We examined different imaging parameters, including a scan rate of 0.2 frame/s, a laser power of 2.5 mW, an acquisition duration of 100 ms, and a total irradiation time of 10 min. Under these imaging conditions, no photomodification was observed across the entire range of wavelengths examined (Supplementary Figure S5). The choice of these parameters was based on their use in imaging *D. discoideum* cells, which are known for fast movement, thus necessitating a high scan rate for effective imaging. The average RMS deviation in this case was found to be 0.043 ± 0.003 μm (Supplementary Figure S6), with no appreciable difference among the results for the different wavelengths. These findings indicate that at the high scan rates that are typically used for fast-moving cells, a wide range of laser wavelengths can be used for live-cell imaging on azopolymer nanotopographies without affecting the features. For slower-moving cells, a lower scan rate is typical for imaging, which is even more advantageous.

**FIGURE 3:**
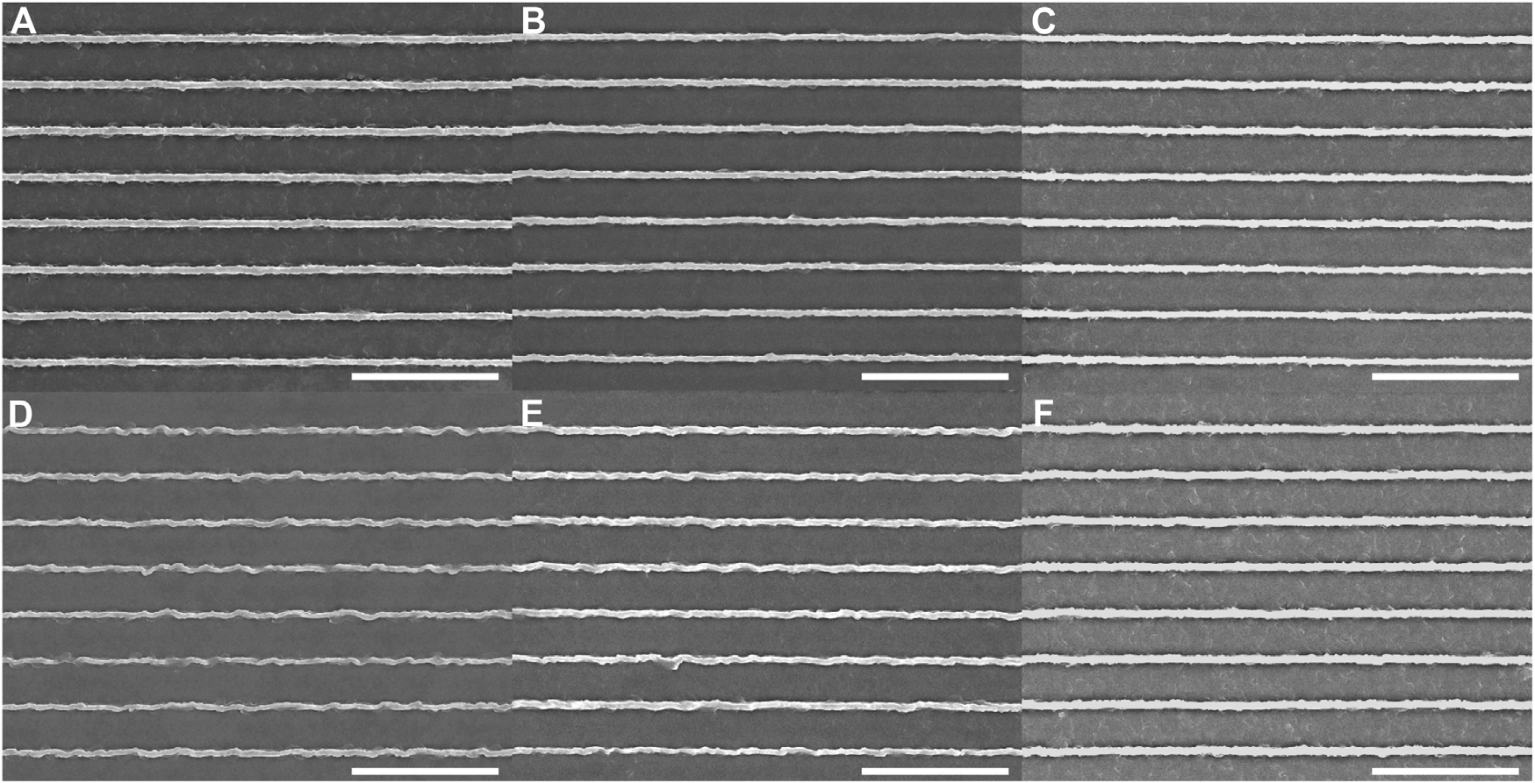
Azopolymer ridges exposed to light at different wavelengths and different powers for 10 min on a spinning-disk confocal microscope: **(A)** 405 nm, 5 mW; **(B)** 440 nm, 4 mW; **(C)** 488 nm, 5 mW; **(D)** 514 nm, 2.5 mW; **(E)** 561 nm, 5 mW; and **(F)** 640 nm, 4 mW. All scans were performed at a rate of 1 frame/s. The image-acquisition time was 1 s. All scale bars are 10 µm.

We also tested a higher frame rate of 1 frame/s, with an exposure time of 1 s and an imaging duration of 10 min. Buckling was not observed following exposure to the 405-nm laser at a power of 5 mW (Figure 3A), to the 440-nm laser at a power of 4 mW (Figure 3B), and to the 488-nm laser at a power of 5 mW (Figure 3C). A modest degree of buckling of the ridges was observed following exposure to the 514-nm laser at a power of 2.5 mW (Figure 3D), and a slight amount of buckling was observed following exposure to the 561-nm laser at a power of 5 mW (Figure 3E). No buckling was observed following exposure to the 640-nm laser at a power of 4 mW (Figure 3F). The average RMS deviation for the ridges (Supplementary Figure S7) after exposure at 405 nm, 440 nm, and 640 nm was 0.044 ± 0.003 μm. Exposure to the 514-nm laser showed the highest RMS deviation of 0.070 ± 0.007 μm. 561-nm exposure led to an RMS deviation of 0.056 ± 0.016 μm. Here, again, the most effective wavelengths for photomodification lead only to minor changes in the pDR1m nanotopography.

Previous work has demonstrated that photoisomerization of azopolymers can occur across a wide range of wavelengths (Yager and Barrett, 2006). Consequently, for live-cell-imaging studies, it has been recommended that laser wavelengths in the red region of the spectrum be used to avoid photomodification of the azopolymer (Rianna et al., 2016; De Martino et al., 2020). This approach represents a significant constraint, in that dyes that are excited in this wavelength range are limited, and it is not possible to perform experiments with multiple different fluorescent labels. For our samples, live-cell imaging can be conducted across a broad range of laser wavelengths without altering the nanotopography at typical maximum scan rates used for imaging *D. discoideum* cells. At the majority of the wavelengths tested, high excitation powers and high frame rates can be used if needed.

The reasons for the differences between our results and previous reports are not entirely clear. One likely factor is that the most efficient switching from the less stable *cis* isomer to the more stable *trans* isomer of pDR1m occurs optically, rather than thermally, which requires the use of a wavelength at which both of these conformers can absorb light. The absorption spectrum typically reported in the literature is that of the *trans* form (Poprawa-Smoluch et al., 2006). There is some evidence in the prior work that the effective overlapping absorption wavelength range for both isomers is in the neighborhood of 530 nm (Poprawa-Smoluch et al., 2006), which is consistent with our results.

Another potential consideration is that previous studies that investigated the wavelength dependence of photomodifiable azopolymers for cell studies focused largely on films of pDR1m, in which mass transport was induced by laser irradiation, resulting in the creation of topographic relief. In contrast, our approach involves the photomodification of existing azopolymer nanopatterns, potentially resulting in different behavior. There is considerably more material in the films used previously than in our nanoridges, and so exposure at any wavelength within the absorption band of the *trans* form may lead to significant heating that can cause molecules in the *cis* form to revert to the *trans* form. The nanoridges contain less material and have greater surface-to-volume ratio than do films, and so may heat substantially less during exposure, thus requiring light to drive the *cis*-to-*trans* transition efficiently. The fact that we observe reciprocity over such a large range of exposure powers supports the idea that heating is not involved substantially in this transition for nanoridges.

### 3.3 2-Photon irradiation studies over a broad range of wavelengths

**FIGURE 4:**
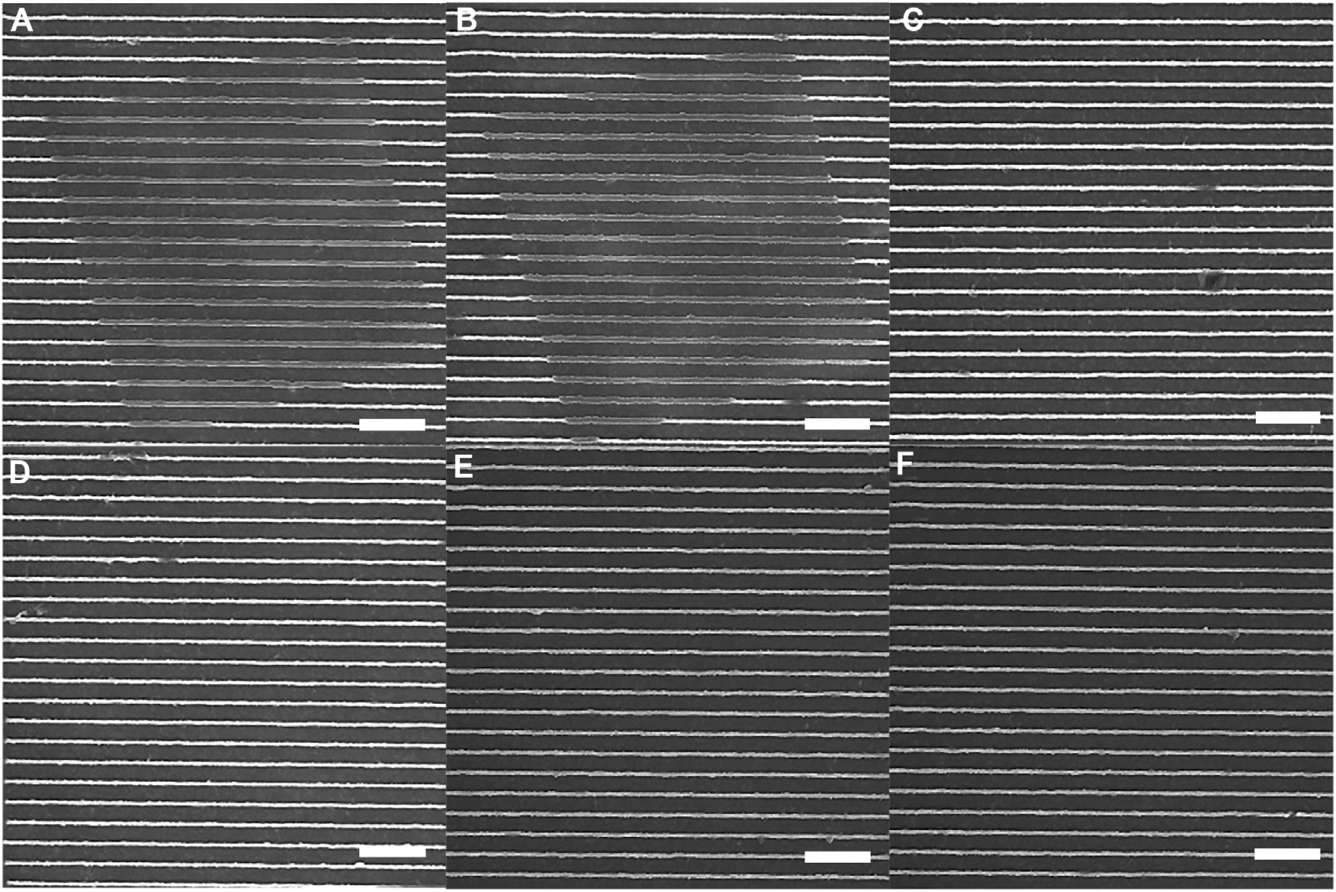
Azopolymer ridges exposed to light at different wavelengths for 10 min on a multiphoton fluorescence microscope: **(A)** 700 nm, **(B)** 725 nm, **(C)** 750 nm, **(D)** 775 nm, **(E)** 800 nm, and **(F)** 900 nm. The light was polarized parallel to the ridges in all cases, and the scans were performed continuously at a rate of 0.5 frames/s. All scale bars are 10 µm.

We next examined the response of azopolymer nanotopography to 2-photon excitation using a multiphoton fluorescence microscope. SEMs of the regions exposed to each wavelength tested are shown in Figure 4. We initially assessed the results of exposure at 700 nm (Figure 4A), 800 nm (Figure 4E), and 900 nm (Figure 4F). Photomodification was only observed in the first case, so we then tested 725-nm (Figure 4B), 750-nm (Figure 4C), and 775-nm (Figure 4D) exposure. Photomodification was observed at 725 nm, but not at the two longer wavelengths. The photomodification is not pure buckling, because the 2-photon absorption tensor need not align with the 1-photon transition dipole of Disperse Red 1.

We conclude from these experiments that multiphoton fluorescence imaging of cells on azopolymer nanotopography can be performed safely at wavelengths between 750 nm and 900 nm. It is likely that the safe exposure range extends further into the infrared, but we did not have an objective with suitable optical transmission at wavelengths longer than 900 nm.

It is somewhat surprising that light at the shortest wavelengths tested here leads to photomodification, given that 1-photon modification occurs most efficiently at a wavelength that would correspond to 2-photon excitation at 1,024 nm. That being said, we did not test 1-photon excitation at wavelengths shorter than 405 nm, so it is possible that 1-photon absorption of light in the 350-nm wavelength range would also lead to photomodification. Another possibility is that 700-nm and 725-nm light are within the tail end of the absorption spectrum of the *cis* isomer of Disperse Red 1, such that the *trans*-to-*cis* conversion is driven by 2-photon absorption but the *cis*-to-*trans* conversion is driven by 1-photon absorption at these wavelengths.

### 3.4 The response of *D. discodium* to *in situ* photomodifiation of nanoridges

Next we explore the feasibility of using two different wavelengths of light, one for live-cell imaging and another for photomodfication of pDR1m nanoridges. To this end, we employed alternating cycles of irradiation with a 514-nm laser at a power of 2.5 mW and a scan rate of 1 frame/s for surface modification, and a 561-nm laser at a power of 3.5 mW and a scan rate of 0.2 frames/s for imaging.

*Dictyostelium discoideum* serves as our model system, representing a challenging scenario in terms of frame-rate requirements, due to the rapid movement and dynamic behavior of these cells. Cells with slower dynamics do not require such a high frame rate, allowing for better performance across a broad range of imaging wavelengths without any signs of photomodification of the underlying azopolymer topographies. Of course, the laser power required for fluorescence imaging depends on the label used and its concentration and/or level of expression.

**FIGURE 5:**
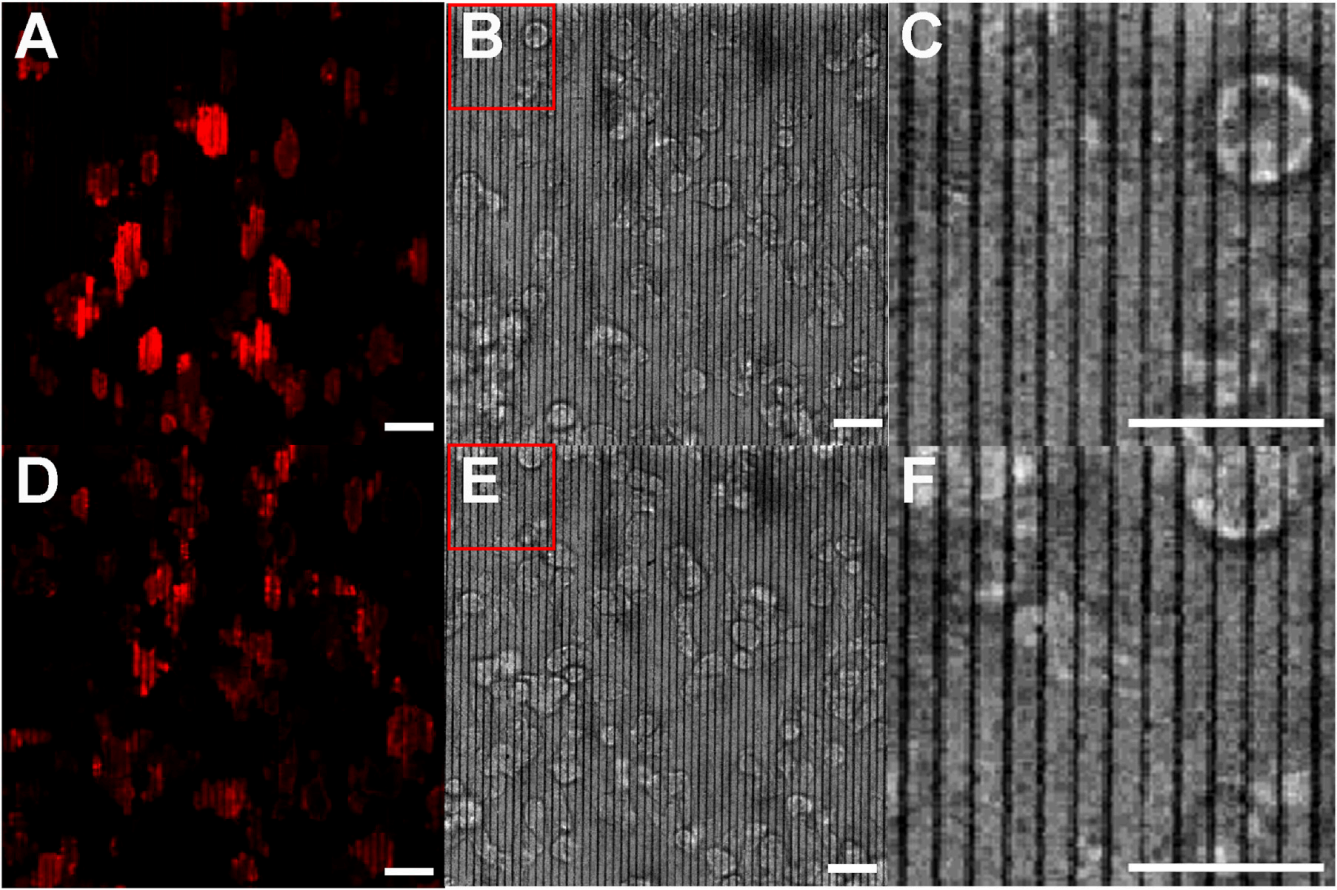
Actin polymerization in *D. discoideum* cells on pristine and photomodified nanoridges. **(A)** Fluorescence micrograph for 561-nm excitation and **(B)** the corresponding bright-field image showing the pristine ridges. **(C)** is a zoom-in of the region indicated in **(B)**. **(D,E)** are images obtained following irradiation with 514-nm light for 10 min while the cells were present. **(F)** is a zoom-in of the indicated region in **(E)**. The zoom-ins indicate that the ridges are not initially wavy, but become so following exposure to 514-nm light. All scale bars are 20 μm.

Figures 5A–C show a fluorescence image under excitation at 561 nm, a bright-field image, and a zoomed-in bright-field image, respectively, of *D. discoideum* cells on pDR1m nanoridges. The nanoridges and actin fluorescence remain unaffected by the exposure. Although the 561-nm laser falls within the effective wavelength range for inducing photoisomerization of pDR1m, the scan rate chosen for this experiment was insufficient to cause photomodification of the nanoridges. Figures 5D–F were captured under identical conditions, but following 10 min of scanning with the 514-nm laser. Notably, the ridges display buckling, accompanied by distinct alterations in actin dynamics. These findings indicate that it is possible to modify nanotopography intentionally in specified patterns and then to perform fluorescence imaging without influencing the nanotopography further, even in the presence of cells featuring multiple fluorescence labels. Moreover, following a total of 55 min of alternating cycles of 561-nm exposure for 10 min and 514-nm exposure for 5 min, no notable photobleaching was observed in the cells.

## 4 Conclusion

We have presented a detailed study of the photomodification of pDR1m nanoridges, particularly under conditions that can be used for live-cell fluorescence imaging. A key result of our studies is that it is possible to perform such imaging under typical conditions over a wide range of wavelengths without affecting the nanotopography. We further found that reciprocity holds for pDR1m over the time scales of typical fluorescence-imaging experiments and beyond. As such, reciprocity can be used in conjunction with the data presented here to assess the likelihood of photomodification of pDR1m nanotopography under other imaging conditions. For instance, in some experiments, such as in studies of stem-cell differentiation or the growth of neural cells, imaging is performed infrequently, but over a period of days or more. Combining our imaging results with the principle of reciprocity can allow for the evaluation of the potential for photomodification of azopolymer nanotopography during such experiments.

A comprehensive evaluation of visible-wavelength irradiation of pDR1m nanoridges was made using a scanning-disk confocal microscope while maintaining constant power, scan rate, exposure time, and imaging duration. The outcomes of these studies reveal that all tested wavelengths did not induce any substantial modification of the nanotopography. At a higher scan rate and laser power, only the 514-nm and 561-nm lasers induced any buckling of ridges whatsoever, with the 514-nm laser having a more pronounced effect. Other laser wavelengths did not result in any photomodification. In contrast, under typical conditions for multiphoton fluorescence imaging, excitation wavelengths between 750 nm and 900 nm were not found to lead to photomodification, but 700-nm and 725-nm excitation did cause photomodification.

Our results stand in contrast to earlier reports. In particular, we find that cells on pDR1m nanotopography can be imaged at any excitation wavelength within the visible range without impacting the nanotopography under a broad range of conditions. Some care must be taken when using 514-nm or 561-nm excitation, but at all of the other wavelengths examined, high excitation powers and frame rates can be used. Perhaps most surprising is the fact that the wavelengths that we tested that were shorter than 561 nm had no effect whatsoever on the nanoridges. We believe that this behavior is a consequence of the fact that heating is not an important channel from the conversion of the *cis* isomer to the *trans* isomer in our system, and that shorter wavelengths of light also cannot drive this transformation.

As a practical demonstration, we used cycles of 561-nm light for imaging and 514-nm light for photomodification of nanoridges in the presence of *D. discoideum* cells. The results of these studies demonstrate the ability to image actin-polymerization dynamics with 561-nm excitation without inducing modification of the nanotopography, and the ability to use the 514-nm laser to modify the nanotopography, and therefore the actin dynamics, without harming the cells.

We note that of the optical imaging methods typically used for living cells, fluorescence imaging generally involves by far the highest exposure to light, and can therefore be considered a worst-case scenario for unintended photomodification of the azopolymer nanotopography. For instance, the wide-field illumination used for bright-field imaging of cells, as in Figure 5, did not lead to any change in the nanotopography.

Our results collectively indicate that the range of potential applications of azopolymer nanotopography in live-cell fluorescence imaging is considerably larger than had been apparent previously. In many instances, the fluorescence of double-labeled or even triple-labeled live cells should be able to be studied without leading to undesired photomodification of the azopolymer nanotopography. At the same time, when desired, photomodification can be accomplished without causing substantial photobleaching of the labeled cells, so long as the fluorophores that are used do not have an absorption maximum near 514 nm.

## Data Availability

The raw data supporting the conclusions of this article will be made available by the authors, without undue reservation.
