# Supplementary material for: Assessing the stability of azopolymer nanotopography during live-cell fluorescence imaging
Source: Front Bioeng Biotechnol. 2024 Aug 13;12:1409735. doi: 10.3389/fbioe.2024.1409735 (PMC11347283; doi:10.3389/fbioe.2024.1409735)
Supplement: Supplementary file 1 [file DataSheet1.docx]

Supplementary Material

## Supplementary Figures


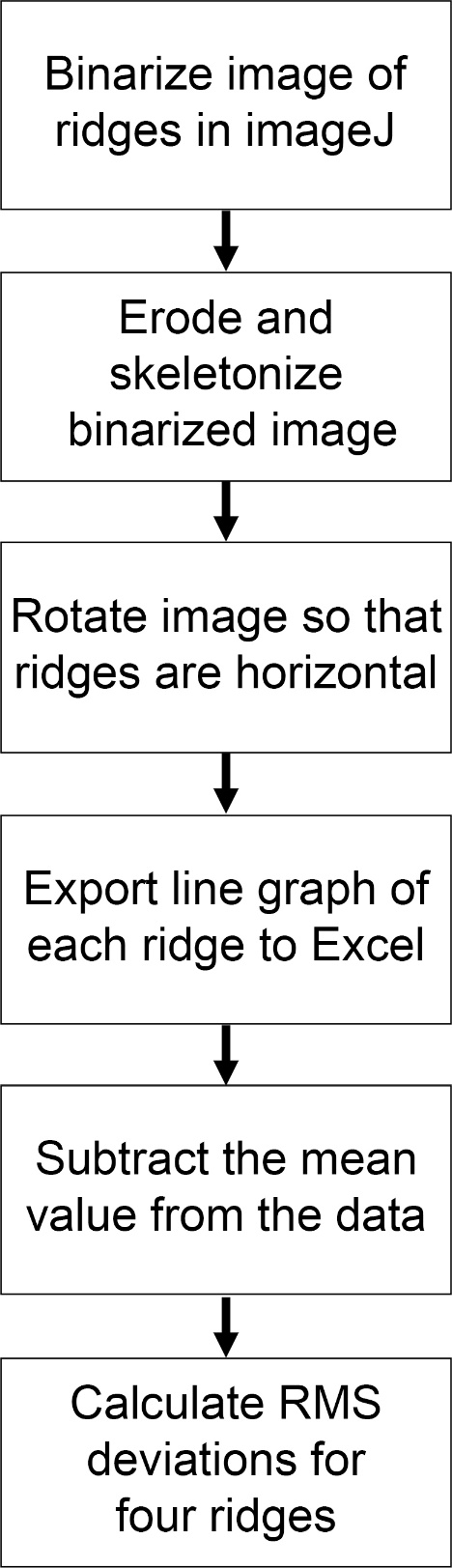


**Supplementary Figure S1.** Workflow for the determination of the RMS deviation of nanoridges.


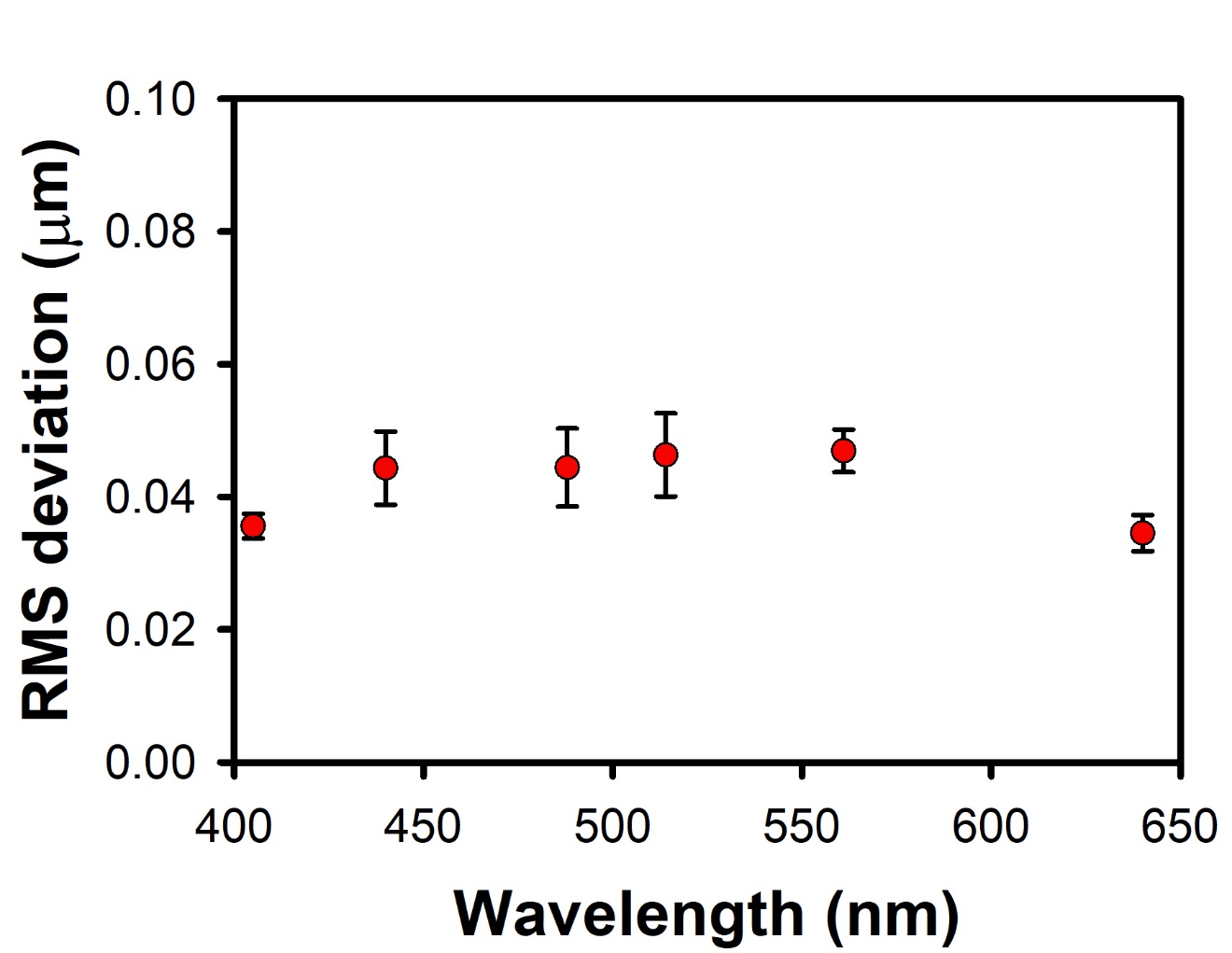


**Supplementary Figure S2.** RMS deviations of the ridges as a function of wavelength following exposure using typical imaging parameters for MCF10A cells for 10 min.


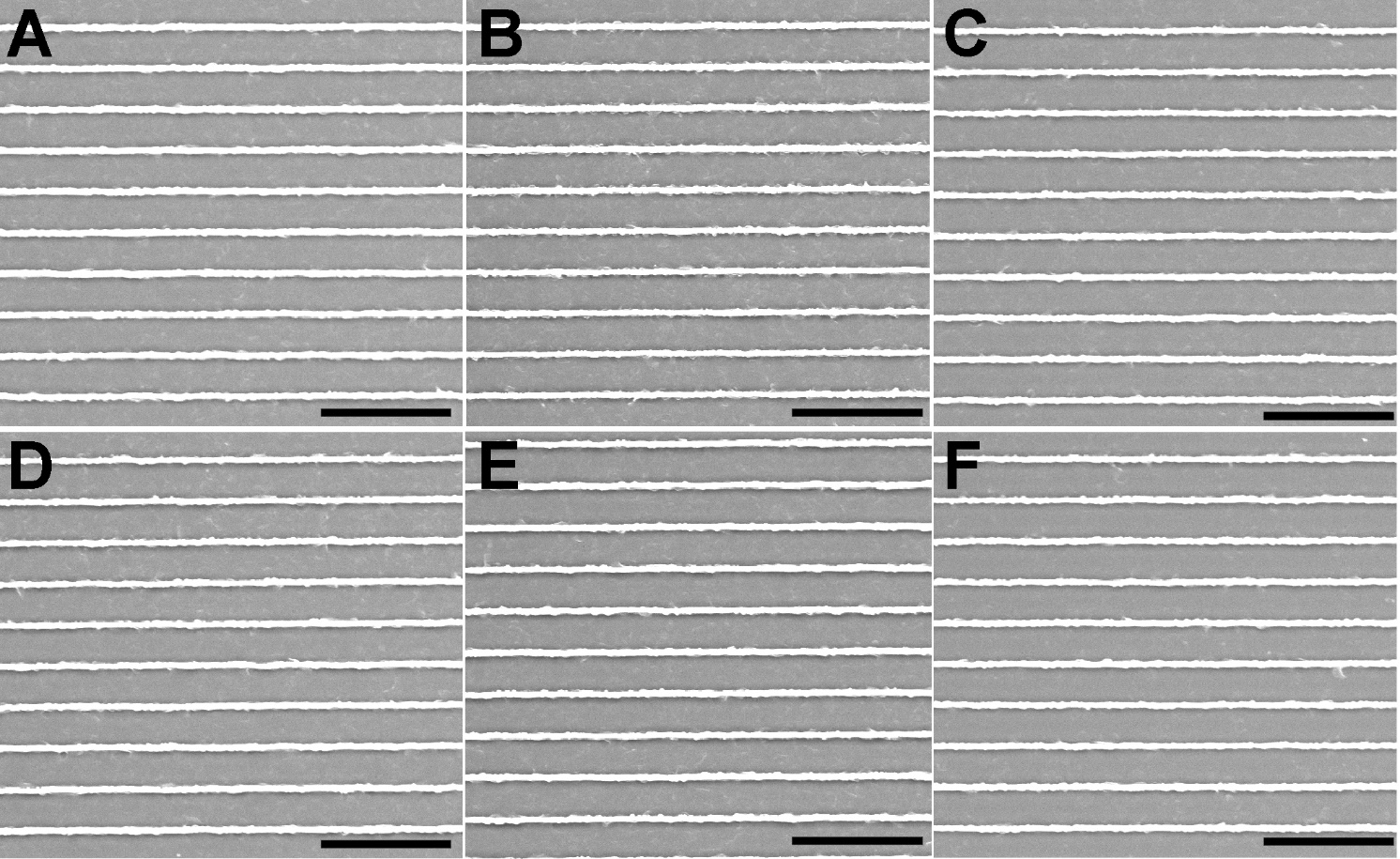


**Supplementary Figure S3.** SEMs of azopolymer ridges exposed to light at different wavelengths and different powers for 30 min on a spinning-disk confocal microscope: (A) 405 nm, 5 mW; (B) 440 nm, 4 mW; (C) 488 nm, 5 mW; (D) 514 nm, 2.5 mW; (E) 561 nm, 5 mW; and (F) 640, 4 mW. All scans were at a rate of 1 frame/sec. The image-acquisition time was 1 s. All scale bars are 10 µm.


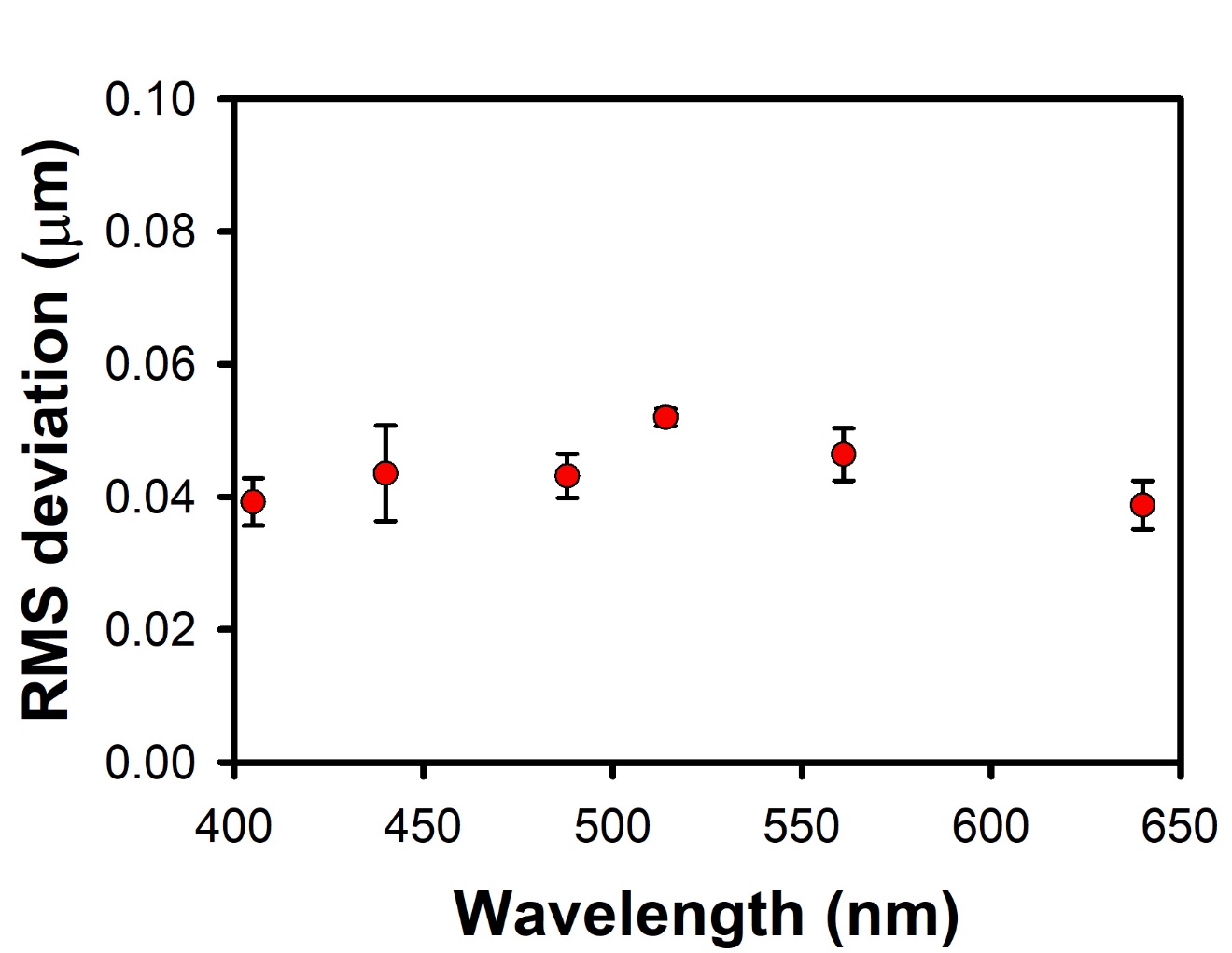


**Supplementary Figure S4.** RMS deviations of the ridges as a function of wavelength following exposure using typical imaging parameters for MCF10A cells for 30 min.


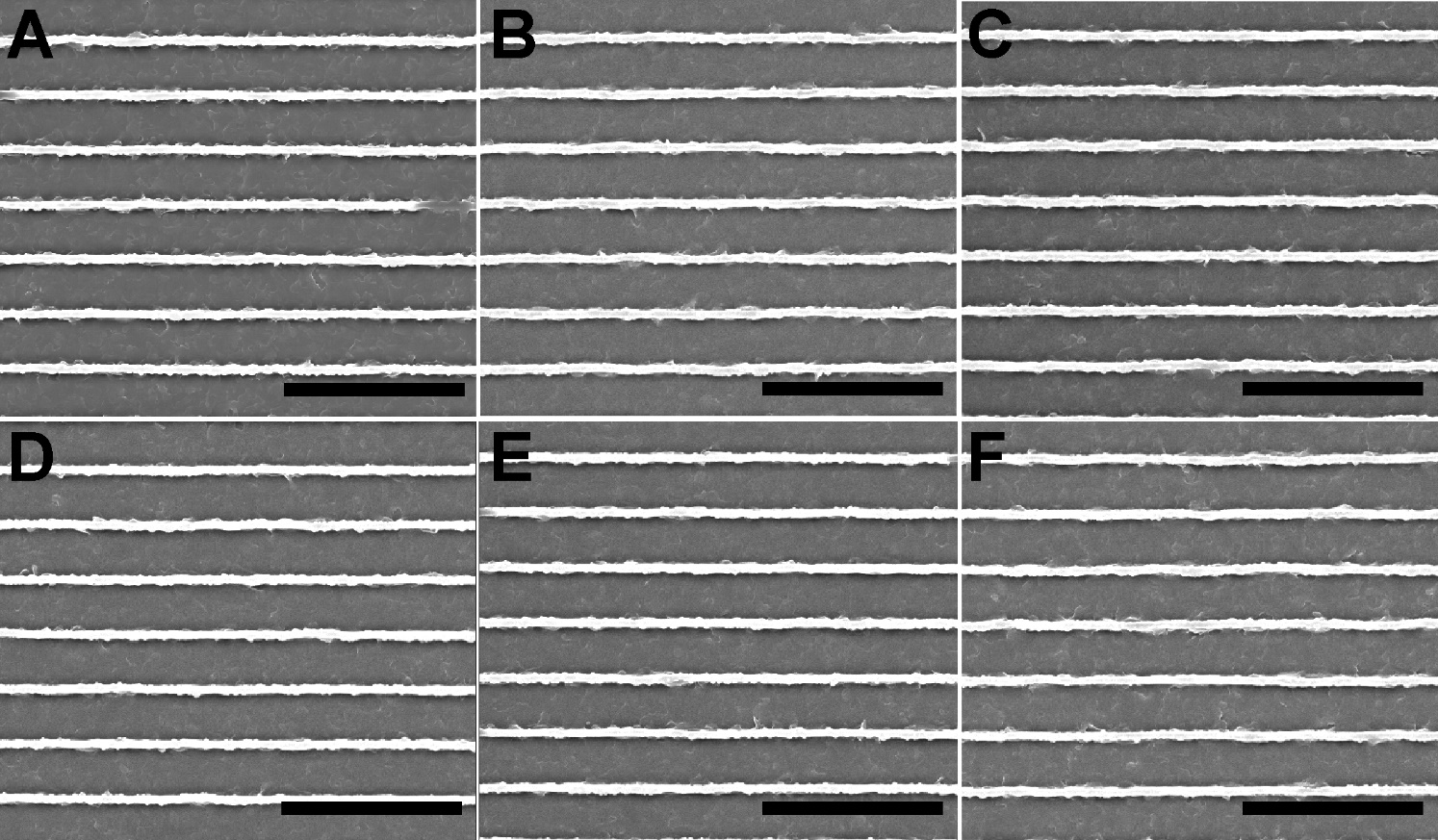


**Supplementary Figure S5.** SEMs of azopolymer ridges exposed to light at wavelengths of (A) 405 nm, (B) 440 nm, (C) 488 nm, (D) 514 nm, (E) 561 nm and (F) 640 nm, for 10 min on a spinning-disk confocal microscope under constant exposure conditions that are typical for live-cell imaging for *D. Discodium cells*. In all cases, the scans were performed at a rate of 1 frame/5 s, the laser power at each wavelength was adjusted to 2.5 mW, and the image-acquisition time was 100 ms. All scale bars are 10 µm.


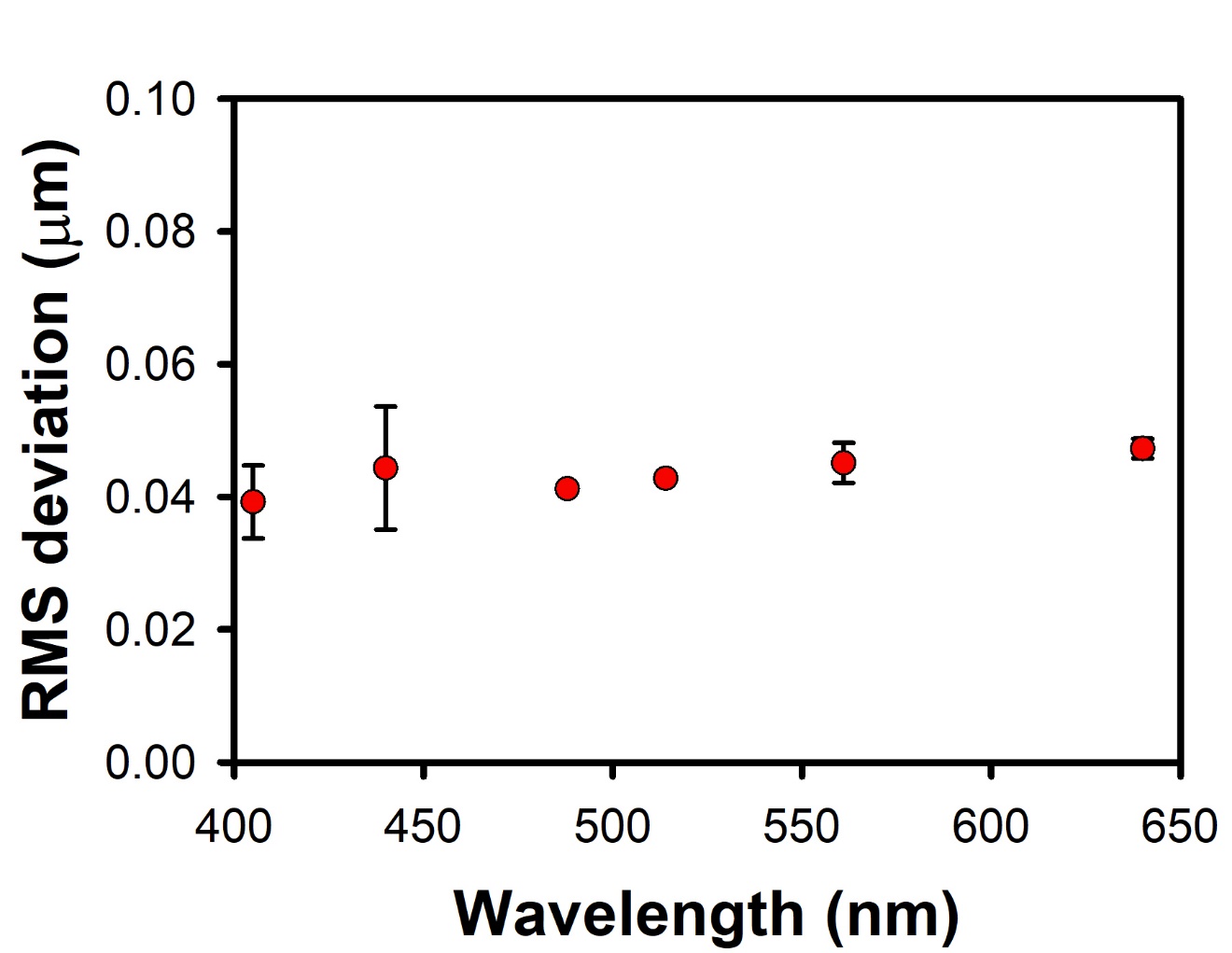


**Supplementary Figure S6.** RMS deviations of nanoridges as a function of wavelength following imaging using typical imaging parameters for *D. discoideum* cells for 10 min.


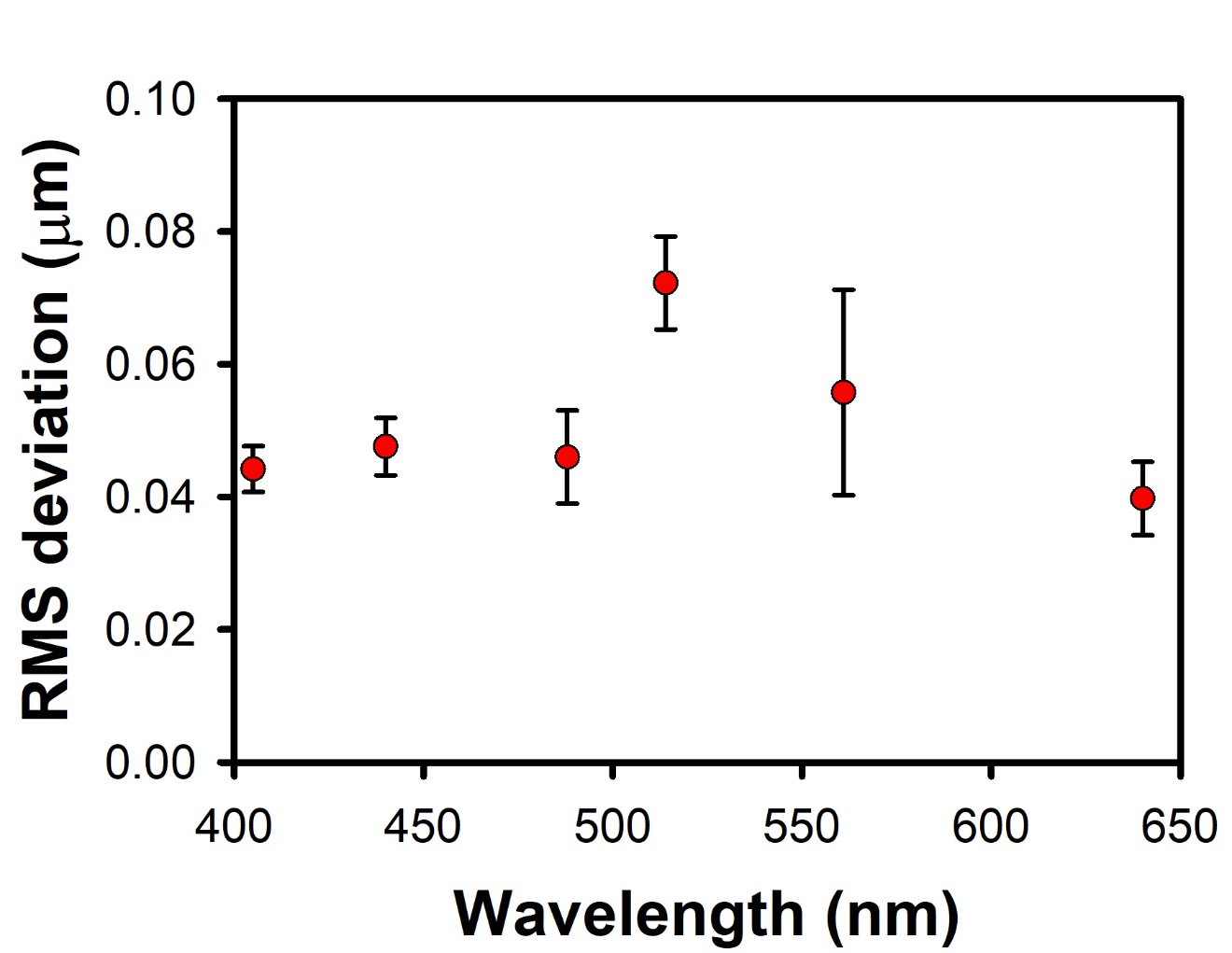


**Supplementary Figure S7.** RMS deviations of nanoridges as a function of wavelength following imaging using high scan-rate parameters.
